# Prevalence, antibiogram and risk factors of thermophilic *campylobacter* spp*.* in dressed porcine carcass of Chitwan, Nepal

**DOI:** 10.1186/1471-2180-14-85

**Published:** 2014-04-05

**Authors:** Laxman Ghimire, Dinesh Kumar Singh, Hom Bahadur Basnet, Rebanta Kumar Bhattarai, Santosh Dhakal, Bishwas Sharma

**Affiliations:** 1 Institute of Agriculture and Animal Science, Rampur Campus, Nepal; and Manager at Dairy Value Chain-Development Project, Dang, Nepal; 2 Department of Pathology and Clinics (HOD), Tribhuvan University, Institute of Agriculture and Animal Science, Rampur Campus, Chitwan, Nepal; 3 Department of Microbiology and Parasitology (HOD), Tribhuvan University, Institute of Agriculture and Animal Science, Rampur Campus, Chitwan, Nepal; 4 The Ohio State University, Columbus, USA; 5 Department of Microbiology and Biochemistry, Himalayan College of Agricultural Sciences and Technology, Kathmandu, Nepal

**Keywords:** *Campylobacter*, Prevalence, Antibiotic resistance, Risk factors, Nepal

## Abstract

**Background:**

*Campylobacter* is the primary cause of food borne gastroenteritis. Moreover, the emergence of multiple drug resistant campylobacters from poultry and pork has produced a potential threat to public health. Research addressing these issues is sparse in Nepal. So, this cross-sectional study aims at determining the prevalence, antibiogram and risk factors of campylobacters from dressed porcine carcass of Chitwan, Nepal.

**Results:**

We collected 139 samples of dressed porcine carcass from 10 different pork shops located in Chitwan district and processed according to OIE Terrestrial Manual, 2008, chapter 2.8.10. Antibiogram of identified *Campylobacter* spp. was evaluated against nine commonly used antibiotics by using disc diffusion method following CLSI guidelines. The prevalence of *Campylobacter* spp*.* was 38.84% (*C. coli* 76% and *C. jejuni* 24%). There was no significant difference (p > 0.05) between the prevalence rate of male (32.4%) and female (41%) carcass. Ampicillin and erythromycin showed the highest resistance (92.59% each) followed by colistin (72.2%), tetracycline (61.1%), nalidixic acid and cotrimoxazole (44.4% each), ciprofloxacin (31.5%) and gentamicin (5.56%). Moreover, 77.8% of the isolates were resistant to more than two antimicrobials. Nalidixic acid and tetracycline showed significant difference (p < 0.05) in the resistivity pattern among different species of Campylobacters. The association between prevalence rate and regular sanitization of slaughter slab equipments was significant (p < 0.05). Similarly, prevalence rate was significantly associated (p < 0.01) with chilling and contamination of intestinal content with carcass.

**Conclusions:**

The pork meat of Chitwan is highly contaminated with antibiotic-resistant Campylobacters and slaughtering practices play significant role in contamination. It is necessary to train the butchers about hygienic slaughtering practice. The consumers as well as butchers should adopt safety measures to prevent themselves from antibiotic resistant campylobacters. The veterinary practitioners should adopt prudent use of antibiotics in pigs.

## Background

*Campylobacter* is the leading cause of bacterial zoonotic gastroenteritis in both developing and developed countries [1]. It causes 2 to 7 times more diarrheal cases than *Salmonella*, *Shigella* or *E. coli* O157:H7 [2]. *C. jejuni* is primarily responsible for human campylobacteriosis. However, the role of *C. coli* cannot be neglected because many studies from Spain and United Kingdom have emphasized the importance of *C. coli* because of its multiple antibiotic resistance property and its ability to cause acquired food borne enteric infections [3,4]. *C. coli* contribute about 9% of human campylobacteriosis in USA [5] and about 7% in England and Wales [6]. *C. coli* cases are even higher than *C. jejuni* in older people [6,7] and in summer [7]. Pork is considered to be the major reservoir of *C. coli*[8]. Various studies have reported *C. coli* as a potential source of human campylobacteriosis. In the United Kingdom, Gillespie et al., showed that individuals harboring *C. coli* infection were more likely to have eaten pork pate than those infected with *C. jejuni*[6]. Similarly, in a large case control study in the USA, Friedman et al., 2004 showed the consumption of hamburgers, pork roasts and sausages as an important risk factor for *Campylobacter* infection [9]. Most of the researches are concentrated on *C. jejuni* and less is explored about *C. coli*[4]. Therefore, this paper focus on prevalence, antibiogram and risk factors associated with *C. coli* in porcine carcass.

Most of the cases of *Campylobacter* infection are self limiting and do not require medication. However, an acute post-infectious ascending paralysis may occur (Guillain-Barr’e syndrome) that is considered most common cause of flaccid paralysis after polio [1]. This condition and severe prolonged infection require treatment. Macrolids and fluroquinolones are drugs of choice for treatment of human campylobacteriosis [10]. However, resistance to these groups of antibiotics have been reported from different part of the world [11,12]. Resistance to fluroquinolones in the treatment of severe cases of human campylobacteriosis has risen in USA since 1990 [13].

Very few studies have been done in Nepal regarding campylobacteriosis. A cohort study was carried out on 77 expatriate adults who had lived in Nepal for <2 years by Shlim et al., 1999 to find out the cause of travelers’ diarrhoea among foreigners in Nepal [14]. Among the causative agents, *Campylobacter* was one of them. He found the annual attack rate of campylobacter as 10%. There are no other available records of human Campylobacteriosis in Nepal. This is probably because most of the cases of Campylobacters go undiagnosed because these cases do not require hospitalization. Moreover, the isolation of *Campylobacter* need sophisticated laboratory and is often time and labor consuming. The consumption rate of pork is increasing in Nepal and at the same time the butchers and consumers are unaware about this issue. In a study carried out by Ghimire *et.al.*, 2013, the condition of pig slaughter slabs was miserable and butchers were unaware about campylobacteriosis [15]. There was high chance of cross-contamination of carcass during slaughtering procedure. So, Nepalese might be at high risk and it is essential to estimate the prevalence of Campylobacters in pork. Antibiotics are widely used in pigs of Nepal for therapeutic and prophylactic purpose [16]. Nepalese people may be constantly consuming antibiotic resistant Campylobacters through pork meat. So, this study is done to determine prevalence, antibiogram and risk factors of *Campylobacter* spp*.* in dressed porcine carcass of Chitwan district.

## Methods

This cross-sectional study was conducted from September 2012 to January 2013.

### Questionnaire and survey

A set of semi-structured comprehensive questionnaire was developed focusing on i) condition of water, ii) sanitization of equipments, iii) slaughterhouse practices and condition, and iv) contamination of carcass with intestinal content. All of the slaughter slabs and retail pork meat shops in Chitwan were visited and butchers were interviewed.

### Sample collection

There are 5 slaughter slabs and 5 retail pork meat shops in Chitwan district. Altogether 139 pooled samples of pork meat (each sample contain meat from neck, ham, shoulder and skin) were collected aseptically from all of these slaughter slabs and retail pork shops in UV sterilized plastic zipped bags and transported immediately to Veterinary Microbiology Laboratory of the IAAS, Rampur in ice cooled box for further processing.

### Bacterial culture

Isolation and identification of thermophilic *Campylobacter* spp. was done according to OIE Terrestrial Manual 2008, chapter 2.8.10. The collected samples were immediately processed without storage. About 10 gm of each samples were mixed with 90 ml 0.1% buffered peptone water (pH 7.2) (M614, HiMedia lab, Mumbai, India) and homogenized manually for pre-enrichment. One volume of homogenized fluid was added to nine volume of Bolton broth (CM0983, Oxoid ltd, Basingstoke, Hampshire, England) for enrichment and then subjected to incubation in microaerophilic atmosphere obtained by burning candle in candle jar (BD1777SE, Don Whitely Scientific Ltd, England) at 37°C for 5 hours and then at 42°C for next 43 hours.

Following incubation, one loopful of broth culture was streaked on modified CCDA (mCCDA) and incubated at 42°C in a microaerophilic atmosphere for 48 hrs in candle jar. When suspected colonies were detected, confirmatory tests including Gram,s stain, growth at 25°C, oxidase and catalase tests, sensitivity to nalidixic acid and cephalothin and hippurate hydrolysis were performed.

### Antibiogram of the isolated species

Antibiogram of identified *Campylobacter* spp. was evaluated against nine different antibiotics (ampicillin, chloramphenicol, ciprofloxacin, nalidixic acid, erythromycin, tetracycline, gentamicin, colistin, and cotrimoxazole) by disc diffusion method following CLSI guidelines. Platinum loop was used to pick pure *Campylobacter* spp*.* colonies from the mCCDA plates and turbid suspension was made by emulsifying colonial growth in BHI broth. The turbidity of the inoculums was adjusted to the equivalent turbidity of 0.5 McFarland standards and the broth was incubated in microphilic condition for 48 hours in anaerobic jar with lighting candle.

After incubation, 100 μl of Brain Heart Infusion broth (M210, HiMedia lab, Mumbai, India) was dispersed over the surface of a Mueller Hinton Agar (MHA) (M173, HiMedia lab, Mumbai, India) with 5% defibrinated sheep blood to produce a lawn of confluent of bacteria on the surface of agar. Using sterile tweezers, antimicrobial discs were placed widely spaced aseptically on the surface of MHA plate. Tweezers were reflamed after application of each disc. The plates were then incubated in microaerophilic condition at 37°C for 24 hours.

Following incubation, the diameter of zone of inhibition was recorded to nearest millimeters for each discs used and then classified as sensitive, intermediate and resistant based on the criteria of Huysmans and Turnidge, 1997 for ampicillin, chloramphenicol, ciprofloxacin, erythromycin, nalidixic acid and tetracycline and for other antimicrobial disc used, CLSI guideline for Enterobacteriaceae was followed. Multiple antibiotic resistance (MAR) was calculated by dividing the total number of antibiotics used by number of antibiotics resistant to particular isolates [17]. In this study, 9 antibiotics were used and are represented as (b), while number of antibiotics resistant to particular isolate is as e.g. 4 (a). MAR is calculated as a/b, which means that in this particular case, MAR is 4/9 = 0.44.

### Statistical analysis

Data entry, management and analysis was done using program Microsoft Office Excel 2007. The association between different risk factors and the antibiotics resistivity pattern of isolated Campylobacters were compared statistically by a Chi-square (χ [2]) analysis using commercial software PHStat2 version 2.5 and Fisher exact test with significance level defined at the p < 0.05. The diameter of zone of inhibition of different antibiotics was compared by using *t*-Test: Two samples assuming equal variances.

## Results

The prevalence rate was found to be 38.85% (54/139). Among the isolates, 42 (77.8%) were *Campylobacter coli* and 12 (22.2%) were *Campylobacter jejuni.* The prevalence rate in male and female carcass is 32.4% (11/34) and 41% (43/105) respectively. The sex-wise prevalence was statistically non-significant (p > 0.05).

**Figure 1:**
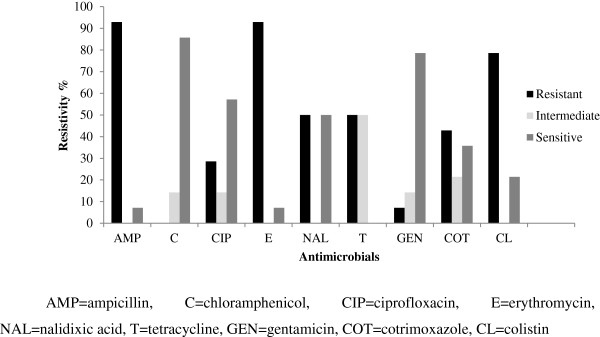
**Antimicrobial sensitivity pattern of *C. coli *** **from dressed porcine carcass.**

**Figure 2:**
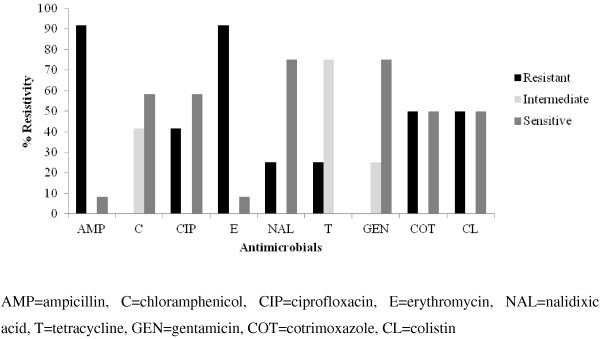
**Antimicrobial sensitivity pattern of *C. jejuni *** **from dressed porcine carcass.**

**Figure 3:**
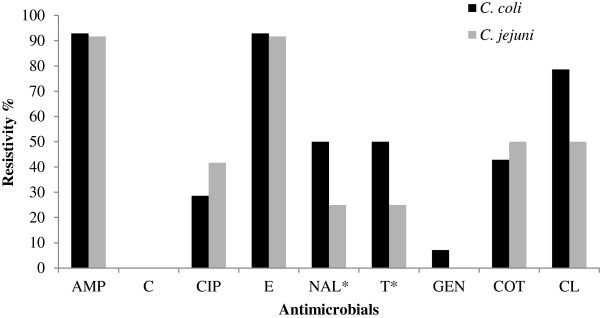
**Antimicrobial resistance pattern of *C. coli *** **and *C. jejuni.***

The antimicrobial sensitivity pattern of *C. coli* and *C. jejuni* is shown in Figures 1 and 2 respectively. The *Campylobacter* spp*.* showed significant (p < 0.05) difference in resistivity pattern with tetracycline and nalidixic acid however, both the species showed similar resistivity pattern with other antimicrobials (Figure 3).

The mean disc diffusion zone among *C. coli* and *C. jejuni* were significantly different (p < 0.01) for chloramphenicol and gentamicin and non significant (p > 0.05) for ciprofloxacin, erythromycin, ampicillin, nalidixic acid, cotrimoxazole, tetracycline and colistin (Table 1).

**Table 1:** **Mean disc diffusion zone diameter for *Campylobacter spp.***

**Antimicrobials**	***C. coli *** **Mean ± SE (mm)**	***C. jejuni *** **Mean ± SE (mm)**	**p-value**
Ampicillin	9.36 ± 0.201	9.17 ± 0.167	p > 0.05
Chloramphenicol	25.50 ± 0.464	21.75 ± 1.232	p < 0.01
Ciprofloxacin	21.43 ± 1.037	20.75 ± 2.125	p > 0.05
Erythromycin	11.14 ± 0.417	10.42 ± 0.417	p > 0.05
Nalidixic acid	15.57 ± 0.996	14.75 ± 0.863	p > 0.05
Tetracycline	18.36 ± 1.078	19.25 ± 1.887	p > 0.05
Gentamicin	16.64 ± 0.467	20.50 ± 1.422	p < 0.01
Cotrimoxazole	15.86 ± 1.167	15.00 ± 1.508	p > 0.05
Colistin	10.79 ± 0.265	11.00 ± 0.302	p > 0.05

MAR index of the isolated *Campylobacter* spp. are shown in Table 2. Every isolates were resistant to at least one of the antimicrobials used in this study. Moreover, 92.6% of the total isolates were resistant to more than one and 77.8% of the isolates were resistant to more than two antibiotics. *C. coli* (85.7%) showed greater multiple antibiotic (more than two) resistance as compared to *C. jejuni* (50%). 22% of the isolates had MAR index between 0.1 and 0.2 and 77.8% of the isolates have MAR index greater than 0.2. The most common multiple antibiotic resistant pattern was ery-amp (85%).

**Table 2:** **Multiple antibiotic resistance (MAR) indices of *C. coli *** **and *C. jejuni***

**MAR index**	**Percentage frequency of MAR index (%)**	
	***C. coli***	***C. jejuni***
0	0	0
0.1	7.1	8.3
0.2	7.1	41.7
0.3	21.4	0
0.4	7.1	8.3
0.5	0	0
0.6	28.6	0
0.7	21.4	41.7
0.8	7.1	0
0.9	0	0
1	0	0

Different factors that influence the prevalence of Campylobacters in pork is shown in Table 3. The prevalence rate was significantly associated with frequency of sanitization of equipments (p < 0.05), contamination of carcass with intestinal content (p < 0.01) and chilling (p < 0.01) (Table 3).

**Table 3:** **Factors influencing prevalence of *Campylobacter *** **spp** ***.***

**Risk factors**	**% of samples examined**	**Prevalence rate**	**p-value**
Sex			
Male	24.46 (34/139)	32.35 (11/34)	p > 0.05
Female	75.54 (105/139)	41 (43/105)	
**Sanitation of equipments**			
Cleaning of Achano*			
Daily	59.7 (83/139)	30.1 (25/83)	p < 0.05
Not daily	40.3 (56/139)	51.8 (29/56)	
Cleaning of weighing machine*			
Daily	30.2 (42/139)	26.1 (11/42)	p < 0.05
Not daily	69.8 (97/139)	44.33 (43/97)	
Contamination of carcass with intestinal content**			
Sometimes	65 (65/100)	64.6 (42/65)	p < 0.01
Never	35 (35/100)	34.3 (12/35)	
Chilling**			
Yes	19.4 (27/139)	3.7 (1/27)	p < 0.01
No	80.6 (112/139)	47.3 (53/112)	

In the above table, *indicates significant at p < 0.05 and **indicates highly significant (p < 0.01).

## Discussion

Campylobacters are regarded as important food borne pathogens. In this study, we found the prevalence of *Campylobacter* spp. in pork meat of 38.85%. This is higher than that previously found in New Zealand (9.1%) [19] and Italy (10.3%) [20], similar to that reported in one 2003 US study (33%) [18], but lower than more recent US study of dressed rib meat (49%) [22] at US. It is also significantly lower than the prevalence rate of 67% found in slaughtered pigs in Tanzania [21].

These differences may be due to slaughtering practices, antibiotic usage, or intrinsic carriage rates. Some of the differences in prevalence rates may also reflect differences in methods used to culture the *Campylobacter*.

This study has also shown higher prevalence rate of *C. coli* than that of *C. jejuni* in pork which is supported by many other research like von Alrock et al. in 2012 (*C. coli* 76% and C*. jejuni* 24%) [23] and Jonker in 2009 (*C. coli* 83.3% and *C. jejuni* 17.7%) [24].

Several studies have shown the occurrence of antimicrobial resistance among *Campylobacter* isolates [25-28]. Most of the isolates in this study (>90%) showed resistance towards ampicillin and erythromycin. This finding is similar to the findings of other investigators in Spain (81.1%) [3] and Denmark (74.4%) [29]. In a study carried out in 2011 in South Africa, Uaboi-Egbenni et al. reported 100% resistance in one farm and 50% resistance in another farm for *C. jejuni* from pig towards erythromycin [12]. In the same study, he reported the resistivity of 100% for *C. coli* in one farm and 64% resistance in another farm towards ampicillin.

Tetracycline showed significant difference in the resistivity pattern between *C. coli* and *C. jejuni*. This finding is in agreement with the findings of Mattheus et al. in 2012 [31]. The resistivity pattern of *C. coli* in this study is in line with Sato et al. and Thakur et al. in 2004 and 2005 respectively [32,33]. Some researchers have shown higher resistivity of tetracycline [3,31]. Nalidixic acid showed significant difference in the resistivity pattern between *C. coli* and *C. jejuni* (*C. coli* being 50% and *C. jejuni* being 25%). Similar to this finding, Mattheus et al. reported the resistivity upto 48.8% in *C. coli* from pigs of Belgium however, he showed decreasing trend of resistivity since 2005 [31].

*C. jejuni* showed higher resistivity (41.7%) than *C. coli* (28.6%) for ciprofloxacin with 31.5% overall resistivity. The result of this study is in line with Gallay et al. in pigs of France [25]. Similarly, Uaboi-Egbenni et al. observed 40% resistance in one of the pig farm in South Africa in 2011 [12] and Mattheus et al. reported the trend of ciprofloxacin resistance in the range of 20% and 48.8% from 2004 to 2009 in Belgium [31]. The overall resistivity is in close association with the reporting of Mattheus et al. in 2012 from pork meat of Belgium [31]. However, higher resistivity has been reported from other parts of Europe (28 to 100%) [3,20]. Fluroquinolones are the drug of choice after erythromycin for the treatment of Campylobacteriosis in human. Therefore, emergence of fluroquinolone resistance is a serious matter of concern and potential threat to public health.

Gentamicin resistance was found low (7.1% in *C. coli* and 0% in *C. jejuni* with 5.5% overall resistivity) in comparison to other antimicrobials used in this study. In a research performed in 2007 in Canada, Norma et al. found 0.2% resistivity against gentamicin [34]. This research has regarded gentamicin and chloramphenicol as safe and effective drugs for the treatment of human campylobacteriosis if pork is considered as the source of infection. However, in-vitro antibiotic sensitivity test should be carried for severe or prolonged or immune compromised cases of food borne campylobacteriosis if the source is unknown.

The prevalence of Campylobacters in chilled and unchilled carcass was statistically significant (p < 0.01). In a study in 1985, Oosterom et al. isolated *Campylobacter* spp*.* from 9% and 0% of the carcasses before and after chilling, respectively [35]. Similarly, in 2008, Nesbakken et al. reported 56.7% and 1.7% prevalence before and after blast freezing of the carcass [36]. Similarly, in 2003, Pearce et al. detected the prevalence rate of 33% in carcass prior to chilling and 0% in chilled carcass [18]. So, lack of chilling the carcass is identified as a risk factor for prevalence of campylobacters in dressed pork.

The prevalence rate in slaughter slab where contamination of carcass with intestinal content occurs sometimes was significantly higher compared to the slaughter slab where such contamination never occurred (p < 0.01). This is due to the fact that the intestinal content of pig is highly contaminated with *Campylobacter*[8,19,30]. So, contamination of carcass with intestinal content is another risk factor for prevalence of campylobacters in pork.

The prevalence of *Campylobacter* spp*.* from slaughter slabs and retail shops where wooden chopping board (*Achano*) was not cleaned daily was significantly higher (p < 0.05) compared to those cleaning the chopping wood (*Achano*) daily. This shows that chopping wood used in slaughter slab could be potential source of *Campylobacter* contamination but samples from these equipments were not cultured for confirmation. So, further research is needed for confirmation. Similarly significant difference (p < 0.05) in the prevalence of *Campylobacter* spp*.* was observed between the pork meat shop that regularly cleaned the weighing machine and others that do not clean weighing machine regularly. So, slaughtering equipments are also risk factors for campylobacter contamination in pork. Oosterom et al. in 1985, ICMSF in 1998 and Pearce et al. in 2003 have also regarded slaughtering equipments as important risk factors for cross contamination of campylobacter in pork [18,35,37].

The MAR index for the isolated campylobacters is very high in this research which is suggestive of public health hazard. All of the isolates are resistant to at least one of the most of commonly used antibiotics included in this study. More importantly, 28.6% of the isolated *C. coli* were resistant to six different antibiotics and 21.4% were resistant to seven different antibiotics used in the study. This implies severe threat to public health. Likewise, 41.7% of the isolated *C. jejuni* were resistant to seven different antibiotics used in the study. The reason behind this may be due to excessive use of antibiotics in pig for treatment as well as growth promoter. The other reason may be due to environmental cross-contamination through other risk factors such as contact with reservoirs like human. This shows that Nepalese people are constantly consuming multiple antibiotic resistant campylobacters in their diet through pork meat. Ery-Amp was the most common resistant pattern (85%) regardless of the species whereas, Thakur and Gebreyes reported ery-tet as most common resistant pattern (60.6%) in porcine carcass from conventional pig rising farms in 2005 [33].

## Conclusions

The pork meat of Chitwan district is highly contaminated with multiple antibiotic resistant thermophilic *Campylobacter* spp*.* in which *C. coli* followed by *C. jejuni* are predominant species. Both the butchers and consumers should be made aware regarding this issue. The isolated Campylobacters showed highest resistivity to macrolids, ampicillin and fluoroquinolones and highest sensitivity to chloramphenicol and gentamicin. So, chloramphenicol and gentamicin should be preferred for the treatment of campylobacteriosis in pigs as well as in human if it is suspected of pig origin. Veterinarians and para-veterinarians should adopt prudent use of antibiotics in pigs. Contamination of intestinal content during slaughtering, cross contamination through slaughter house equipments and lack of chilling facilities are the major risk factors of *Campylobacter* contamination. Routine monitoring of slaughter slab condition and strict implementation of Animal Slaughter and Meat Inspection Act 2055 should be done together with the awareness campaign for the butchers and consumers.

## Competing interests

None of the authors have any competing interests.

## Authors’ contributions

LG participated in study design, bacterial culture, data analysis and drafting manuscript, DKS participated in data analysis and bacterial culture identification, HBB participated in bacterial culture and identification, antibiogram and drafting manuscript, RKB conducted bacterial culture, antibiogram and assisted in drafting manuscript, SD participated in data analysis and interpretation, survey of butchers and manuscript preparation and BS participated in bacterial culture, survey of butchers and drafting manuscript. All the authors read and approved the final manuscript.
